# Understanding the influence of all nodes in a network

**DOI:** 10.1038/srep08665

**Published:** 2015-03-02

**Authors:** Glenn Lawyer

**Affiliations:** 1 Max Planck Institute for Informatics, Campus E1 4, 66123 Saarbrucken, Germany

## Abstract

Centrality measures such as the degree, k-shell, or eigenvalue centrality can identify a network's most influential nodes, but are rarely usefully accurate in quantifying the spreading power of the vast majority of nodes which are not highly influential. The spreading power of all network nodes is better explained by considering, from a continuous-time epidemiological perspective, the distribution of the force of infection each node generates. The resulting metric, the *expected force*, accurately quantifies node spreading power under all primary epidemiological models across a wide range of archetypical human contact networks. When node power is low, influence is a function of neighbor degree. As power increases, a node's own degree becomes more important. The strength of this relationship is modulated by network structure, being more pronounced in narrow, dense networks typical of social networking and weakening in broader, looser association networks such as the Internet. The expected force can be computed independently for individual nodes, making it applicable for networks whose adjacency matrix is dynamic, not well specified, or overwhelmingly large.

Networks have become the premier approach to describing spreading processes such as epidemics or information transfer because they express the heterogeneity of interactions characteristic of many human activities^1^. Thirty years of innovation have refined our ability to identify nodes which are highly influential to the outcome of almost any spreading process on a given network via features such as betweenness centrality^2^,^3^, eigenvalue centrality^4^, degree^5^, or k-shell^6^. Yet highly influential nodes are rare by definition, and the just listed measures are not informative for the vast majority of network nodes. These centrality measures only rank nodes and are not designed to quantify spreading power^6^,^7^,^8^. While the rankings accurately identify the few highly influential nodes, they can considerably underestimate the spreading power of non-hub nodes^9^. Nor do these rankings explicitly incorporate the dynamics of spreading processes^10^,^11^. This leaves open the question of quantifying the spreading power of the vastly more numerous non-highly influential nodes, and indeed understanding the nature of node spreading power itself. As highly influential nodes only rarely originate spreading processes, be they pathogenic disease^12^,^13^, innovative ideas^14^, or chatter^15^, there is deep intellectual hunger and practical utility in accurately measuring and understanding the spreading power of each individual node in a network.

A node's spreading power is the force with which it can push a spreading process to the rest of the network. This definition can be made more precise by reference to the common epidemiological models of spread. In a susceptible-infected (SI) spreading process without recovery, which inevitably reaches the entire connected component of the network, the spreading power of the seed node predicts the delay before half (or some other large percentage of) the network is reached. In a process with recovery to either the susceptible (SIS) or immune (SIR) state, spreading power correlates to the probability that a node can seed an epidemic given that the ratio of the per-contact transmission rate to the rate of recovery allows for, but does not guarantee, an epidemic. When this ratio exceeds the critical range, the dynamics approach the SI system as a limiting case.

Several approaches to quantifying the spreading power of all nodes have recently been proposed, including the *accessibility*^16^,^17^, the *dynamic influence*^11^, and the *impact*^8^. These extend earlier approaches to measuring centrality by explicitly incorporating the dynamics of spread. The accessibility is a modified form of hierarchical degree which controls for both transmission probabilities and the diversity of walks of a given fixed length^17^. The dynamic influence, like the eigenvalue centrality, is the proportion of infinite walks starting from each node, where walk steps are scaled such that the linear dynamics of the system are expected to converge to a non-null steady state^11^. The impact sums, over increasing walk lengths, the probability of transmission to the end node of the walk and that the end node has not been previously visited by a shorter walk^8^. These new spreading power metrics have been shown to be distinct from previous centrality measures and more highly correlated with epidemic outcomes^8^,^11^,^18^. Yet they retain the common foundation of the more usual approaches to centrality, counting walks on the network^10^,^19^,^20^,^21^. As the walks are counted using powers of the adjacency matrix, spread is observed only in discrete time.

Epidemiology, in contrast, studies the continuous-time dynamics of the force of infection (FoI), defined as the current rate at which susceptible nodes are becoming infected^22^. In network models, the FoI is directly proportional to the current number of edges between infected and susceptible nodes. The critical distinction between FoI and walks is that the FoI is determined by the number of infected-susceptible edges, independent of their distance from the seed node. The critical distinction between continuous- and discrete-time is that continuous-time allows resolution down to the first two transmissions, a level not easily expressed in a discrete-time framework where multiple transmissions may occur at each time step. The distinction is acute, as the number of events per time step grows at a double-exponential rate in scale-free networks^23^, the type of network most representative of human structures^24^ and perhaps even life itself^25^.

The continuous-time epidemiological perspective suggests that node spreading power can be accurately quantified by appropriately summarising the distribution of the number of infected-susceptible edges after a small number of transmission events arising from a seed node in an otherwise fully susceptible network; that is, by the expected FoI generated by that node. We here propose such a measure, named the *expected force* (ExF), and show that it outperforms the accessibility, k-shell, and eigenvalue centrality in predicting epidemic outcomes in SI, SIS, and SIR spreading processes, in both discrete- and continuous-time. The basis in local neighborhood structure means the ExF is applicable even when the full adjacency matrix is either unknown or inherently unknowable. The metric naturally extends to weighted and directed networks. Most importantly, the expected force is able to illuminate the factors responsible for node spreading power.

## Results

### Definition of the Expected Force

**Figure 1:**
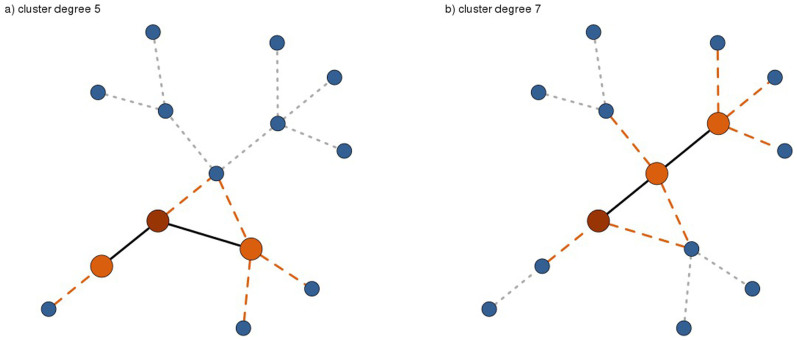
Deriving the expected force from the possible outcomes of two transmissions. This network will be in one of eight possible states after two transmissions from the the seed node (red). Two states are illustrated, where the seed has transmitted to the two orange nodes along the solid black edges. Each state has an associated number of (dashed orange) edges to susceptible nodes (blue), the cluster degree. States containing two neighbors of the seed (panel a) can form in two ways or, if they are part of a triangle, four ways. The eight network states associated with the pictured seed node arrise from thirteen possible transmission clusters. The expected force of a seed node is the entropy of the distribution of the (normalized) cluster degree over all (here 13) possible transmission clusters.

The expected force is a node property derived from local network topology, independent of the rest of the network or any specific spreading process. It is formally defined as follows. Consider a network with one infected node *i* and all remaining nodes susceptible. Enumerate all possible clusters 1, …, *J* of infected nodes after *x* transmission events, assuming no recovery (See Figure 1). Generally speaking, *x* = 2 is sufficient and assumed for the rest of this manuscript. Hence *J* includes all possible combinations of *i* plus two nodes at distance one from *i*, and *i* plus one node at distance one and one at distance two. The enumeration is over all possible orderings of the transmission events. Two neighbors of the seed (*a* and *b*) form two clusters ([*i* → *a*, *i* → *b*] and [*i* → *b*, *i* → *a*]) or, if *a* and *b* also share an edge, four clusters. After two transmissions without recovery, the FoI of a spreading process seeded from node *i* is a discrete random variable taking a value in (*d*_1_, …, *d_J_*), allowing for the proportionality constant equal to the transmission rate of the process. The expected force of infection can be approximated by the entropy of the *d_j_* after normalisation 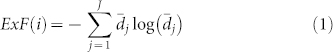
where *i* refers to the seed node and 
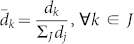
, ∀*k* ∈ *J*.

The entropy is needed for generating the expected value due to the extreme variability in the shape, number of modes, and number of terms in the distributions of *d_j_* for different seed nodes. Complex networks have scale-free degree distributions. The moments of scale-free distributions are divergent, implying that the distribution of *d_j_* may not have a mean value in the traditional sense. The entropy is a standard tool for taming unruly distributions due to its close relation to cumulant generating functions, motivating the use of Equation 1 to generate a quasi-expected value of the FoI. A loose analogy can be made to the use of entropy in statistical physics to summarise the macrostate of a system (e.g. the pressure of a gas) based on the distribution of its microstates (the positions and momentums of molecules in the gas). The analogy is that pressure is a combination of the number and the heat of the molecules, likewise, a node's expected force is a combination of the number of possible transmission clusters it can form and the FoI generated by each cluster. An in-depth discussion of the relationship between entropy, cumulants, and statistical physics can be found in Touchette's review^26^.

Setting *x* = 2 is recommended but not required. Supplementary investigations show that increasing the number of transmissions beyond two adds very little information while increasing the computational cost (see Supplementary Note 1), in agreement with other proposed spreading power metrics^8^,^11^ and consistent with the decaying influence of longer paths in the calculations of the eigenvalue, subgraph, and related centralities^4^,^7^,^20^,^21^. In certain cases, however, it may be desirable to consider more transmission events. For example, a node at the end of a chain of length two can only form one transmission cluster of size two, hence its expected force is zero. Comparing two such nodes requires setting *x* = 3, in which case a subscript can be used for clarity (e.g. *ExF*_3_).

One modification may be in order for SIS/SIR processes, inspired by the following. Imagine a node with degree one connected to a hub. While such a node will have a high expected force, its chance of realizing this force depends entirely on transmitting to the hub before recovery. Such nodes are common in dense social networks. For example, 84% of the 225K nodes in an EU institution email network^27^ have degree one. In such networks, it may be helpful to account for the dependence on the initial transmission by multiplying the ExF by the log of the seed node's degree after first rescaling the seed's degree by some factor *α* > 1. 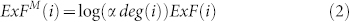
The rescaling is motivated in that the log of one is zero, and the ExF*^M^* is most informative in networks where many nodes have degree one. The rescaling factor must be greater than one, and should also be small to avoid overpowering the influence of the degree. In the rest of this manuscript, we use *α* = 2, the smallest integer which satisfies these criteria. Supplementary Note 2 shows that computing the ExF*^M^* for *α* ranging from 1.0001 to 16 does not substantively alter the metric, as all such variations show correlations greater than 0.99 to ExF*^M^* computed with *α* = 2.

Straightforward calculation of the expected force has time complexity 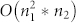, where *n*_1_ and *n*_2_ are the number of neighbors at distance one and two from the seed. It is difficult to analytically compare a time complexity computed on individual nodes with time complexities whose calculation is based on the entire adjacency matrix. Further, since the metric relies only on local information, it can be computed in a massively parallel fashion, or only computed on nodes of interest. It also allows meaningful (partial) computations even on massive graphs, i.e. those whose size overwhelms computer memory. Nonetheless, some comparison to run-times of existing metrics is required. We benchmark the median run time over fifty Pareto networks of 1,000 nodes for all measures discussed here. Run time on each network is measured as the median computation time over ten runs on that network, with computation time measured at sub-microsecond accuracy^28^. Computing the ExF for all non-hub nodes takes 0.16 seconds. The k-shell is computed in 2% of that time (0.003 seconds), and the eigenvalue centrality in 20% of that time (0.03 seconds). Computing the accessibility takes several hundred times longer. The benchmarking is then repeated with the same protocol on 10,000 node Pareto networks. Increases in running time for the k-shell (6x), eigenvalue centrality (9x), and expected force (16x) have roughly linear correspondence to the tenfold increase in the number of network nodes. Recall that the proven time complexity for the k-shell and expected time for the eigenvalue centrality are both *O*(|*V*| + |*E*|), i.e. linear. As expected, the accessibility does not scale well, with a ten-fold increase in network size leading to a 265 fold increase in median running time. Recall that it is computed by taking powers of the adjacency matrix, i.e. something worse than *O*(|*V*|^2.4^). Benchmarking was performed within the R^29^ programming environment running on a commodity laptop computer. K-shell and eigenvalue computations are computed via standard functions in the Igraph package^30^. The accessibility is computed in native R^29^ code using sparse matrix multiplication from the Matrix package 1.0-10^31^. The expected force is computed in C code via an R interface.

Example code providing an implementation of the expected force is available at https://github.com/glennlawyer/ExpectedForce.

### Correlation to epidemic outcomes

We measure correlations between expected force and epidemic outcomes on five families of simulated networks chosen such that their densities and degree distributions span a wide range of human contact structures, as listed in Table 1. One hundred random networks of 1,000 nodes are generated in each family. Further comparison is made using a suite of twenty four real-world networks ranging from 1,133 to 855,800 nodes, as listed in Table 2. Epidemic outcomes are the time to half coverage for SI processes and the epidemic potential for SIS and SIR processes. These are observed by simulating multiple epidemics in both continuous and discrete time from a number of seed nodes in each network. Correlations are measured between these outcomes and the expected force, ExF*^M^*, accessibility, eigenvalue centrality, and the k-shell of the seed nodes. Motivations for these choices and additional details are given in the methods.

**Figure 2:**
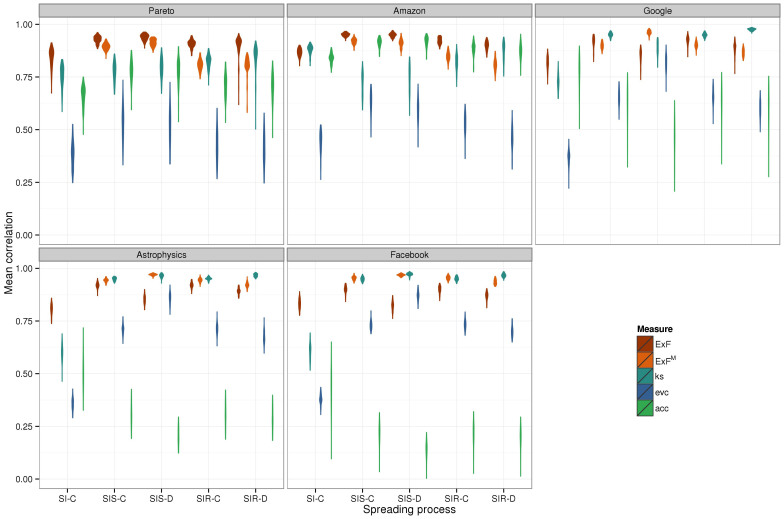
Correlation of spreading power metrics to epidemic outcomes on simulated networks. Violin plots show the distribution of observed correlation values for each spreading process outcome in each network family. The expected force and ExF*^M^* (orange shades) are consistently strong, with mean correlations greater than 0.85 and small variance. The other measures (k-shell, eigenvalue centrality, and accessibility, blue-green shades) show both lower mean values and higher variance, as seen in the position and vertical spread of their violins. Each violin summarizes correlations computed on 100 simulated networks. Spreading processes (x axis) are suffixed to indicate simulations in continuous (-C) or discrete (-D) time. The epidemic outcome for SI processes is the time until half the network is infected. For SIS and SIR processes it is the probability that an epidemic is observed.

**Figure 3:**
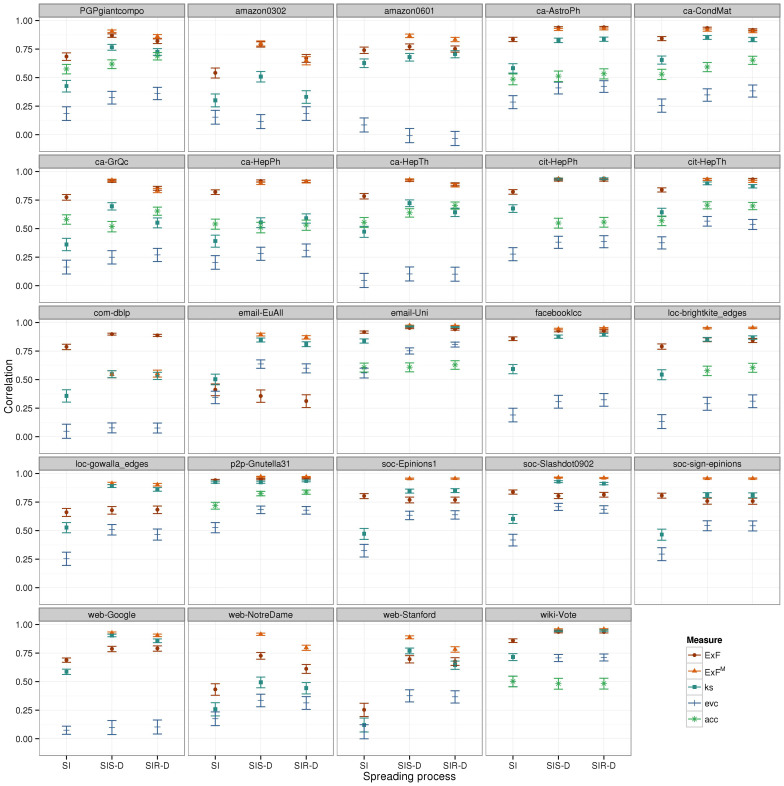
Correlation of spreading power metrics to epidemic outcomes on real networks. Point and error bar plots show the observed correlation and 95% confidence interval between each measure and spreading process outcome on the 24 real networks. The expected force and ExF*^M^* (orange shades) show strong performance, consistently outperforming the other metrics (k-shell, eigenvalue centrality, and accessibility when computed, blue-green shades). The epidemic outcome for SI processes is the time until half the network is infected. For SIS and SIR processes it is the probability that an epidemic is observed. The suffix “-D” indicates spreading processes simulated in discrete time. Individual panels are given as separate (larger) figures in Supplementary Figures 1-6.

The expected force is highly predictive of all epidemic outcomes on all networks tested, simulated and real. Mean correlation to SI process outcomes is 83% on simulated and 74% on real networks. For processes with recovery, mean correlation is 91% on simulated and 82% on real networks. Standard deviations over the one hundred simulated networks in each family are typically 0.02–0.03. The 95% confidence bounds on real networks are in the same range. In all cases the ExF (or ExF*^M^*) significantly outperforms the accessibility and the eigenvalue centrality (difference in mean correlations greater than the standard deviation of the higher mean). It typically outperforms the k-shell, significantly outperforming it in 82 cases, showing equivalent performance in 11 cases (difference in mean correlations less than the standard deviation of the higher mean), and significantly lower performance in 6 cases (simulated Internet networks SIS-C, SIR-C, SIR-D; simulated Astrophysics networks SIR-D, simulated Facebook networks SIR-D, “email-EUAll” network SI). The performance of the k-shell was surprisingly strong, given that two previous studies by independent groups have observed rather poor performance for this metric^11^,^18^. The observed correlations on 100 simulated networks in each family are shown in violin plots (Figure 2); the information is duplicated in tabular form in Supplementary Table 5. Likewise, the measured correlations and their standard errors for all real networks are shown in Figure 3, given in tabular form in Supplementary Tables 6, 7 and 8, and plotted individually in Supplementary Figures 1-6.

The expected force's predictive power is robust to variation in network structure. The theory behind the ExF*^M^* suggests that the ExF might lose performance for SIS/SIR processes on denser networks, yet mean correlation for continuous time SIS processes is barely changed between the loose Pareto/Amazon networks (0.93/0.95) and the dense Astrophysics/Facebook networks (0.92/0.90). As expected, the predictive power of the ExF*^M^* improves on the denser networks (mean correlations: Pareto/Amazon 0.89/0.92, Astrophysics/Facebook 0.94/0.95). The accuracy of the accessibility metric, in contrast, collapses for all spreading processes on the dense networks (mean correlation over all spreading processes: Pareto/Amazon 0.74/0.90, Astrophysics/Facebook 0.28/0.20.) A previous analysis which observed similar poor performance for the accessibility on dense networks concluded that spreading processes seeded from nodes with low accessibility are not capable of entering the epidemic phase^18^. Our results show this is not the case, as these nodes have a small yet observable epidemic potential which the expected force is able to capture and quantify. Performance of the k-shell and the eigenvalue centrality is also strongly influenced by network structure. For SIS/SIR processes, both showed higher mean and sharply reduced variance on the denser networks. In an interesting contrast, the k-shell's predictive power for SI processes is reduced in denser networks. The eigenvalue centrality's performance also varies by spreading process, showing its best performance on discrete time SIS models– though again this variation is modulated by network density. Two other independent groups have observed that relationships between centrality rankings and epidemic outcomes are strongly influenced by network structure and the parameters of the spreading processes^8^,^9^, leading the authors of ref. 9 to conclude that these measures severely underestimate the epidemic impact of structurally peripheral nodes.

### Weighted graphs

The expected force generalizes to graphs with weighted edges, where we assume the edge weights correspond to per-edge transmission likelihoods. Use these weights to calculate the probability of each way that each cluster could occur, and re-define the cluster degree as the sum of all edge weights leading out from that cluster. The extension to directed graphs is also straightforward; limit the enumeration to edges leading from an infected to a susceptible node.

We test this generalization by computing the weighted and unweighted expected force on 1,000 node networks with Pareto (1,2.3) degree distributions and edge weights chosen according to one of the following three distributions: uniformly distributed between one and three, uniformly distributed between one and ten, and exponentially distributed with unit rate, weights rounded up to the nearest integer. Fifty networks were simulated for each distribution of edge weights. Correlation between the weighted and unweighted ExF was greater than 0.99 for all network edge weighting distributions tested. As expected from the tight correlation, the weighted and unweighted ExF showed no meaningful difference in predictive ability, which remained high. Observed correlations between node expected force and epidemic potential in discrete-time SIS processes were 0.88/0.89 ± 0.03 (unweighted/weighted ExF) under the uniform-3 scheme, 0.83/0.04 ± 0.03 under the uniform-10 scheme, and 0.80/0.79 ± 0.05 under the exponentially distributed weighting scheme.

## Discussion

The expected force predicts all types of epidemic outcomes with high accuracy over a broad range of network structures and spreading processes. The low variance in observed correlations over multiple simulated network and epidemic models shows that the measure is robust, as do the tight confidence bounds on real world networks. What, then, does it tell us about the nature of node spreading power? The definition of the expected force implies that spreading power is determined by both the degree of the node and the degree of its neighbors, and that the relative influence of these two factors is different for nodes of low versus high spreading power. Weaker nodes gain what strength they have from their neighbors, whereas more influential nodes get their strength from their large number of connections. These relationships are accentuated by network density.

**Figure 4:**
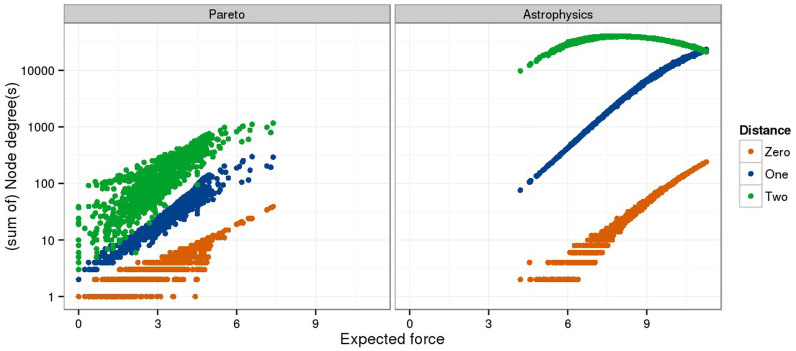
Spreading power is a factor of a node's first and second order degree. Plotting expected force (x-axis) versus node degree (orange), the sum of the degree of all neighbors (blue), and the sum of the degree of all neighbors at distance 2 (green) shows that for nodes with low ExF, the neighbor's degree has strong correlation to ExF, while for nodes with high ExF their own degree is more closely correlated. The result is accentuated in denser collaboration networks in comparison to more diffuse Pareto networks. Correlation between ExF and neighbor degree is 0.94 ± 0.01 in collaboration networks, and drops to 0.84 ± 0.02 in Pareto networks (mean taken over 50 networks; See Supplementary Table 3 for the correlations over all network structures).

This is a result of the combinatorics behind the enumeration over transmission clusters. The number of paths with one edge (*p*_1_) contributes quadratically to the number of transmission clusters, while the number of two-edge paths (*p*_2_) contriutes linearly, since *J* = *p*_1_ * (*p*_1_ − 1) + *p*_2_. Node degree is exactly *p*_1_. Neighbor degree is at most *p*_2_. Weaker nodes tend to have lower degree, hence neighbor degree contributes more heavily to their expected force. The influence of network density comes in part from the ExF's sensitivity to network motifs such as triangles and squares. Each triangle is traced by two paths with two edges, increasing the proportion of *p*_2_ associated with node degree. More importantly, the ExF is the entropy of the onward connectivity of each transmission cluster. A triangle generates four such clusters, each of which has identical cluster degree. Likewise, each square represents two clusters. These network motifs, which are more common towards the cores of dense networks, reduce the disparity of the cluster degree distributions thus increasing entropy. The combinatorics become more complicated when the enumeration is based on more than two transmissions, but these general patterns remain. These relationships can be seen by plotting ExF against the sums of the degrees of nodes at increasing geodesic distance from the seed (Figure 4, Supplementary Table 3).

The approach taken by the expected force is fundamentally different than that taken by most centrality measures. Centrality measures typically set out to produce a ranking which identifies the most influential nodes in the network, under the assumption that highly influential nodes are those with the maximal sum of some type of walk^8^,^10^,^19^,^20^,^21^. The choice of the appropriate type, scaling, and length of walks contain implicit assumptions regarding network flows^10^, cohesion structure^19^, and/or other topological characteristics^20^,^21^ of the network. The k-shell is a slight exception, as it was originally intended to precipitate out the most cohesive regions of the network rather than to explicitly rank nodes within cohesive regions^32^, yet it is now recognized as one of the best centrality measures for identifying a network's most influential spreaders^6^. Spreading power metrics generalize the walk counting framework by explicitly including transmission probabilities when scaling the walks^8^,^11^,^16^,^17^. The question not asked is if the type, scaling, and lengths of walks best suited to identifying the most important nodes applies equally well to the rest of the network. To the extent that the optimal choice of factors depends on network topology, then the difference in topology between core and periphery suggests that choices well suited to the core are less appropriate for the remainder of the network.

Both the combinatorics behind the expected force and the walk counting behind most centrality measures agree that influential nodes are those which combine high degree with a preponderance of influential neighbors. The ExF has high rank correlation with both the eigenvalue centrality and the k-shell (0.62–0.92 across the simulated network families, see Supplementary Note 3). Likewise, the ExF has 60–90% agreement with the eigenvalue centrality on the top ten network nodes and 100% agreement with the k-shell. The difference between walk counting and the expected force is that the expected force adopts the relative influence of different walks and walk lengths based on local connectivity, whereas approaches based on functions of the adjacency matrix apply a fixed protocol. The eigenvalue centrality is weighted node degree, where the weights are the importance of the neighbors^4^,^7^. But the eigenvalue centrality is strictly a global measure, unable to distinguish more subtle variations in local structure^7^,^21^. The k-shell erodes node degree to match the number of neighbors with similar degree. Since this discards remaining information on the individual degree of nodes within a common shell, the accuracy of its predictions is heavily influenced by the number of shells in the network. The accessibility combines node and neighbor degree into a measure of the number of nodes likely to be reached by walks of a given length^17^. But this approach has difficulties quantifying nodes in dense, small diameter networks, which accentuate differences between core and peripheral topology.

The expected force offers additional advantages over existing spreading power and centrality measures in that its calculation depends only on the local topology. This allows epidemic outcomes on the whole network to be predicted with high accuracy even when only a small portion of the network is known. It is rare for the full structure of a real network to be fully known; typically the network structure is inferred from indirect, incomplete, and often biased observations. Specification of an adjacency matrix is even more difficult when the underlying network is dynamic. These limits have practical implications. Estimates of eigenvalue centrality fluctuate depending on which nodes are sampled^33^. Both the pagerank^34^ and the k-shell^35^ are highly sensitive to pertubations in network topology, making them unreliable for incomplete or noisy systems.

Reliance on a local neighborhood is consistent with established theory showing that topological information content falls off quickly with distance. Bonacich demonstrated in 1987 that the eigenvalue centrality can be expressed in terms of sums over walks of length *k*, *k* = 1 … ∞, establishing that the influence of walks must decay at least exponentially in *k* to guarantee convergence^4^. More recent work shows that almost all centrality measures, including those based on matrix resolvents, can likewise be expressed as infinite sums over walks, and that decay rates faster than exponential are often motivated^20^,^21^. The fall-off in information can also be shown by the following example. Consider a long linear chain of nodes which ultimately connects to a network hub. Let *β* be the transmission/recovery ratio in a process with recovery and Δ*_i_* the distance from the *i^th^* node of the chain to the hub. If the spreading process reaches the hub, an epidemic is almost certain. The probability of this occuring is at best 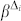. For *β* < 0.1, this probability is estimatable to three of four decimal places using only local information. More generally, since epidemic spread is almost instantaneous on scale free networks^23^,^36^, the expectation is that the time-step which takes a process outside the local neighborhood of its origin brings it to the majority of the network.

Reliance on a local network does, however, lead to one weakness in the expected force. A network may contain large but disparate communities. Here, a node serving as a bridge between two communities might be able to spread a process to the entire network with more force than a node far from the bridge, even when the second node has more (local) spreading power than the bridging node. The expected force's local nature makes it blind to these larger topological constraints on spread.

This work defines the epidemic outcome in SIS/SIR processes as the probability that an epidemic occurs. This is in contrast to the measure typically used, the mean number of infected nodes (i.e. refs. 6, 8, 9, 11, 17, 18, 37). We are not convinced that the mean is a good summary statistic. In over 20,000 simulated continuous-time SIS spreading processes, no processes which went extinct reached more than 20 nodes, while processes which did not go extinct reached the majority of the network. It has been argued that such bifurcation in outcomes is predicted by theory^38^. Given that the distribution of the number of infected nodes is characterized by two well separated modes, the mean is best seen as an indirect estimate of the likelihood of the higher mode. It is this likelihood which we directly measure as the epidemic potential.

The expected force predicts epidemic outcomes from local features of specific nodes on a specific network, with only passing reference to the nature and parameters of the spreading process. Seminal work has approached the question from the other side. Given the parameters of a SIR spreading process and a class of networks characterized by its degree distribution, exact solutions for typical values of a number of epidemic outcomes are available^37^, and their time course can be expressed as paired ordinary differential equations^39^. Node spreading power can be thought of as explaining that portion of the variance around these typical values which is due to the choice of seed node.

The expected force is strongly correlated to epidemic outcome, outperforming existing metrics of node spreading power and centrality. The measure depends only on local network topology, allowing its use in dynamic as well as static networks. For most nodes, the most important determinant of their spreading power is the sum of their neighbors' degree. As node power grows, so does the importance of the node's own degree. This relationship is accentuated in denser networks.

## Methods

### Comparisons

The predictive power of the expected force is compared to one spreading power metric and two centrality measures. Spreading power metrics are a recent theme in the centrality literature specifically designed to quantify the spreading power of all nodes. They have consistently been shown to have better correlations with epidemic outcomes than previous centrality measures^8^,^11^,^18^. The accessibility is chosen to represent them as it is the most general, most powerful, and most theoretically developed existing spreading power metric. In contrast, the authors of the dynamic influence write that their metric is only accurate when the actual transmission probability is close to the assumed optimal value contained in their method^11^. The impact is not defined for SI models, has a different definition for SIS than for SIR models, and directly depends on the transmission probability of a specific spreading process^8^.

Comparison is also made to traditional centrality measures. With the understanding that almost all of these are based on sums of walks of various lengths^10^,^19^,^20^,^21^, and that the best choice for walk length is influenced by network topology^8^,^9^,^19^, we here make comparison to measures which represent both ends of the spectrum. The eigenvalue centrality counts the number of infinite walks, and degree counts walks of length one. We here compare to the k-shell instead of degree, as the k-shell can be thought of as the minimal degree of a node^32^, and is a better measure of node influence^6^. No comparison is made to subgraph centrality as it is computationally too expensive to compute for large graphs^30^. No comparison is made to pagerank because its rankings are highly dependent on the damping factor, including frequent rank reversals after small changes^40^,^41^, and as it is also unstable in the face of small pertubations in network topology^34^.

The crux of the comparisons is this: For each network, a set of seed nodes is selected. Then, for each seed node, the outcome of each spreading process is noted. The correlation between the observed outcome and each metric is measured over all seed nodes for the given network. The eigenvalue centrality and the k-shell are computed using the Igraph package version 0.6.5^30^. We implement the latest version of the accessibility^17^ in R, following python code supplied by the authors and using sparse matrix multiplication from the Matrix package version 1.0-10^31^.

Seed nodes for the simulated networks are selected as follows. For each network, the ExF is computed for all non-hub nodes, with hubs defined as nodes whose degree is greater than 60% of the maximum degree node in the network. The range of observed ExF values is split into 15 equal width bins. Five seed nodes are selected uniformly at random from each bin, giving approximately 75 seed nodes for each of the hundred simulated networks in each of the five network families. The reason for the binning is that in scale free networks, the distribution of any centrality measure is strongly biased towards low values. Selecting seed nodes across the full range of the ExF ensures that nodes with high and medium ExF are also included in all tests. That hubs have maximal spreading power is already firmly estalished, and also the reasons why; they also have the highest ExF values. Further, under this definition, less than 0.1% of nodes are hubs.

### Epidemic simulations and outcomes

Spreading processes without recovery will eventually cover the entire network. The epidemic outcome is the time to half coverage (*tthc*); the time until exactly half the nodes in the network are infected. This is measured at each seed node by simulating 100 spreading processes and fitting the observed *tthc* from all simulations to a gamma distribution. Simulations are run in continuous time, with the time of the next transmission event drawn from an exponential distribution with rate equal to the current number of infected-susceptible edges. The exponential distribution, which models the distribution of waiting times until the next event in a Poisson process, is standard for such simulations as it is memoryless. These simulations are conducted only in continuous time as discrete time does not provide sufficient resolution for SI processes.

Spreading processes with recovery are of interest when the ratio of transmissibility to recovery for the process *β* is in the critical range which allows for but does not guarantee an epidemic. The epidemic outcome is a node's epidemic potential (*EPo*), the probability that a given node can seed an epidemic. The *EPo* is measured by simulating 100 outbreaks per seed node and counting the number which result in at least half of the network nodes becoming infected. Continous-time simulations model the time of the next transmission as in the SI model just described, except the transmission rate parameter scales the current number of infected-susceptile edges by some *β* in the critical range (see below), and the time of the next recovery from a unit rate exponential distribution weighted by the number of infected individuals. In each round of the discrete-time simulations, transmission occurs along each infected-susceptible edge with probability *r* = −log(1 − *β*) (to convert the continous time rate into a discrete time probability) and nodes recovering at the end of the round.

The critical range for *β* can be defined empirically using the criterion that if *β* is in the critical range, then a large majority of nodes will have *EPo* ∈ [2%, 98%]. We here set *β* independently for each network to a fixed multiple of 1/*λ*, where *λ* is the largest eigenvalue of the adjacency matrix. Similarity in network structure allows the same multiple to be used for both the Pareto and Amazon networks, likewise the Astrophysics and Facebook networks. Though the Internet lies between these two other classes, the multiples from the social networks yields good results. This proceedure resulted in at least 96% of nodes in continuous-time simulations with *EPo* in the critical range; in discrete time the relevant figure is 76% or better. Supplementary Table 4 gives the multiples used and the percentage of nodes fulfilling the criterion for all simulated networks and spreading processes.

### The networks

The five families of simulated networks are defined by their degree distributions, one theoretical (Pareto), and four derived from the following real-world human contact networks: the Amazon co-purchase network from May 2003^42^, the graph of the Internet from the 2002 Google programming contest^43^, the collaboration network from ArXiv Astrophysics between 1993 and 2003^27^, and Facebook wall posts from the New Orleans network^44^. The Pareto and Amazon networks are characterized by large diameter and low density. The Astrophysics and Facebook networks are two orders of magnitude more dense and have correspondingly smaller diameter. Google's map of the Internet lies in between the other two families. The networks can also be characterized by the largest eigenvalue of their adjacency matrix, as theory suggests that the critical disease transmission probability dividing epidemic from extinction regimes is the inverse of this value^11^, which further implies that a network's inherent susceptibility to disease spreading is reflected by this eigenvalue. Again, the selected network families cover a wide range of inherent susceptibility to disease. The networks used for the simulations are characterised in Table 1. Simulations are conducted using giant components of 1,000 nodes. Networks with a Pareto (1,2.3) degree distribution are simulated using the Chung Lu protocol, a versatile and general method for constructing random graphs with given expected degree sequence^45^. The remaining networks are simulated by randomly sampling 1,000 values from the degree sequence of the actual graph without replacement and generating a graph from these values using the Igraph function “degree.sequence.game” which generates undirected, connected simple graphs matching the input degree sequence^30^.

Simulated networks allow testing on multiple networks of the same type. This is critical, in that the templates used to create the simulations are themselves static snapshots of dynamic processes. Multiple simulations give some indication of the underlying probability space of the network family. The disadvantage is that the simulated networks model only the degree distribution of the base network, ignoring higher order structure such as communities. In our defense, we note that even defining network community structure remains an open problem, let alone replicating it. Further, certain measures of community structure suggest that the size and composition of commmunities is a factor of network size^43^ implying that it is impossible for a simulated network with 1K nodes to replicate the community structure of i.e. the 876K nodes in Google's map of the internet.

Real world networks were selected and downloaded from the Stanford Large Network Repository (SNAP) and Alex Arenas's collection according to the following criteria: having between 1,000 and 1,000,000 nodes in the largest connencted component, representing one clear network structure, and, in the case that the same network is sampled at multiple timepoints, the latest timepoint meeting the other criteria. Twenty one networks from SNAP and two from Alex Arena passed these criteria. The simulated Amazon networks are derived from the earliest Amazon co-purchase network in SNAP. For completeness, this network is also included in the suite of real networks. The Facebook network was downloaded from the Max Planck Institute for Software Systems. For the purpose of testing, networks are treated as undirected graphs with multiple and self-edges removed. All twenty four real networks are characterized in Table 2, which includes the internet address of the collections.

The size of the real world networks required some slight modifications to the overall approach. Seed nodes are 1,000 nodes selected uniformly at random. Epidemics with recovery are simulated only in discrete time. As can be seen from the results on the random networks, discrete time simulations provide approximately the same mean outcome as continuous time, and only slightly higher variance. The transmission/recovery probability ratio *β* is determined independently for each network by binary search over possible values until one is found such that a minimum of 80% of tested nodes have *EPo* between 0.05 and 0.95. When the network has more than 25,000 nodes, the *tthc* is measured as the time of the 1,000*^th^* transmission rather than the time when half the network is infected. Finally, the R software package does not gracefully handle the multiplication of matrices larger than 25Kx25K, even with the Matrix package^31^. Hence the accessibility was not computed for networks with more than 25k nodes.

## Author Contributions

Glenn Lawyer designed the project, developed the method, performed all reseach, and is solely responsible for the final manuscript.

## Supplementary Material

Supplementary Information Supplementary Information

## Figures and Tables

**Table 1:** Simulated network families. The mean diameter, mean graph density, and empirical middle 65% quantile range of the largest eigenvalue for the different network families. Pareto and Amazon co-purchase networks have a large, loose structure with low eigenvalue, suggesting less inherent susceptibility to epidemics than the smaller and more dense collaboration networks; Google's map of the Internet lies in between. Means and standard deviations are computed over 100 simulated networks with 1,000 nodes

	diameter	density	65% quantile
Pareto	11.6 ± 1.0	3.2 e-04	7.1–10.1
Amazon [42]	7.2 ± 0.4	6.9 e-04	10.1–13.7
Internet [42]	7.0 ± 0.5	9.4 e-03	25.2–35.2
Astrophysics [27]	5.5 ± 0.6	2.1 e-02	54.5–61.9
Facebook [44]	5.5 ± 0.5	2.4 e-02	65.2–73.7

**Table 2:** Real world networks. The number of nodes, 90*^th^* percentile effective diameter, and density of the real networks. Networks were downloaded from the Stanford Large Network Collection (SNAP), Alex Arena's collection (AA), and the Max Planck Institute for Software Systems website (MPI), which in turn credit the cited publication for the network

	nodes	diameter	density	source
PGPgiantcompo	10680	10.0	4.26 e-4	AA [46]
amazon0302	262111	11.1	0.26 e-4	SNAP [42]
amazon0601	403364	7.6	0.30 e-4	SNAP [42]
ca-AstroPh	17903	5.0	12.30 e-4	SNAP [27]
ca-CondMat	21363	6.5	4.01 e-4	SNAP [27]
ca-GrQc	4158	7.6	15.53 e-4	SNAP [27]
ca-HepPh	11204	5.8	18.74 e-4	SNAP [27]
ca-HepTh	8638	7.4	6.65 e-4	SNAP [27]
cit-HepPh	34401	5.0	7.11 e-4	SNAP [47]
cit-HepTh	27400	5.3	9.38 e-4	SNAP [47]
com-dblp	317080	8.0	0.21 e-4	SNAP [48]
email-EuAll	224832	4.5	0.13 e-4	SNAP [27]
email-Uni	1133	4.3	85.00 e-4	AA [49]
facebooklcc	59691	5.6	4.09 e-4	MPI [44]
loc-brightkite	56739	6.0	1.32 e-4	SNAP [50]
loc-gowalla	196591	5.7	0.49 e-4	SNAP [50]
p2p-Gnutella31	62561	6.7	0.76 e-4	SNAP [27]
soc-Epinions1	75877	5.0	1.41 e-4	SNAP [51]
soc-Slashdot0902	82168	4.7	1.49 e-4	SNAP [43]
soc-sign-epinions	119130	4.9	0.99 e-4	SNAP [52]
web-Google	855802	8.1	0.12 e-4	SNAP [43]
web-NotreDame	325729	9.4	0.21 e-4	SNAP [53]
web-Stanford	255265	9.7	0.60 e-4	SNAP [43]
wiki-Vote	7066	3.8	40.36 e-4	SNAP [52]

SNAP http://snap.stanford.edu/data/index.html.

AA http://deim.urv.cat/aarenas/data/welcome.htm.

MPI http://socialnetworks.mpi-sws.org/data-wosn2009.html.
