# Supplementary material for: Understanding the influence of all nodes in a network
Source: Sci Rep. 2015 Mar 2;5:8665. doi: 10.1038/srep08665 (PMC4345333; doi:10.1038/srep08665)
Supplement: Supplementary Information [file srep08665-s1.pdf]

Supplementary materials for “Understanding the influence of all nodes in a network.”

Glenn Lawyer
Max Planck Institute for Informatics

Supplementary Notes 1-3 and Tables 1-3 present further information on topics mentioned in the main text. The remaining tables (4-7) and figures (1-6) present the data from the main text in more detail.

Supplementary Note 1: Correlation between $\mathbf{ExF_2}$ and $\mathbf{ExF_3}$. The Expected Force is based on the distribution of the force of infection after an arbitrary number of infection events; a subscript can be used to indicate the number of events considered. Evidence that two events is sufficient is provided by the tight correlation between the metric when computed using two and again with three events (ExF_2 and ExF_3) for the simulated network classes considered here. The mean and standard deviations in the correlations, taken over 50 networks in each class, are as follows: Pareto 0.96 $\pm$ 0.007, Amazon 0.95 $\pm$ 0.013, Internet 0.97 $\pm$ 0.007, Facebook 0.99 $\pm$ 0.014, Astrophysics 0.99 $\pm$ 0.005. As expected from these tight correlations, increasing the number of events to three does not provide any meaningful increase in predictive accuracy.

Supplementary Note 2: Invariance of $\mathbf{ExF^M}$ to choice of scaling parameter. The modified version of the expected force is defined in the main text as follows:

$$ExF^M(i) = \log(\alpha\, deg(i))\, ExF(i)$$

where the degree of the node is scaled by α so as to prevent the logarithm from being zero for nodes with degree one. A simple shuffling of terms clarifies the influence of α: $ExF^M = \log(\alpha)ExF(i) + \log(deg(i))ExF(i)$, implying that as $\alpha \to 1$, the scaling factor becomes irrelevant, and as $\alpha \to \infty$, it eclipses any contribution from the degree. The manuscript suggest $\alpha = 2$ is a reasonable choice, providing the needed scaling without unduly skewing the measure.

We here show that the measure is largely invariant to the choice of α by testing the following values: 1.0001, 1.001, 1.01, 1.1, 1.5, 2, 3, 4, 8, and 16. For each α tested, the correlation between ExF^M at $\alpha = 2$ and the test value is measured. Measurements are made on all non-hub nodes for one hundred networks of each of the five simulated network families. The mean value for each parameter/network type is reported in Supplementary Table 1. We test over the full network as this is likely to bias the testing values towards low degree nodes, where the choice of α is more likely to have an effect.

All correlations are greater than 0.999 for α in the range 1.5–3. Only two cases show correlation less than 0.99, both occurring when $\alpha = 16$. If instead of reporting the mean correlation observed over the one hundred networks, we report the minimum, the same patterns hold, with the lowest value dropping to 0.976, again for $\alpha = 16$.

Supplementary Note 3: Agreement between the expected force, k-shell, and eigenvalue centrality on the most important nodes. We here assess the agreement between the ExF, k-shell, and eigenvalue centrality as to which nodes are the most important in the network. All three measures are compared on one hundred networks for each of the five families. Supplementary Table 2 shows the mean rank correlation between ExF and the other measures, as well as the agreement between ExF and eigenvalue centrality regarding the top ten nodes.

Overlap with the k-shell is problematic in that the k-shell does not provide deep resolution. In the looser networks, the highest k-shell contains a large percentage of the total

nodes in the network (mean 41% in Pareto, 92% in Amazon). Even in the denser networks, the top k-shell contains more than 10% of the network nodes (Internet 15%, Facebook 13%, Astrophysics 14%). Hence the observation that the top 10 nodes (1% of the network) as ranked by the ExF are also found in the highest k-shell is not sufficiently meaningful to report in the table.

Supplementary Table 1. Correlation between $\mathrm{ExF}^{\mathrm{M}}$ computed with scaling parameter 2 and with the value given in the column headings.

	1.0001	1.001	1.01	1.1	1.5
Pareto	0.9958	0.9959	0.9960	0.9971	0.9994
Amazon	0.9980	0.9980	0.9981	0.9986	0.9997
Internet	0.9965	0.9965	0.9966	0.9975	0.9995
Facebook	0.9995	0.9995	0.9995	0.9996	0.9999
Astrophysics	0.9996	0.9996	0.9996	0.9997	0.9999
	2	3	4	8	16
Pareto	1.0000	0.9992	0.9980	0.9942	0.9899
Amazon	1.0000	0.9996	0.9989	0.9966	0.9939
Internet	1.0000	0.9992	0.9979	0.9931	0.9873
Facebook	1.0000	0.9999	0.9996	0.9987	0.9975
Astrophysics	1.0000	0.9999	0.9997	0.9989	0.9979

Supplementary Table 2. Pearson's rank correlation coefficient between the Expected Force and the eigenvalue centrality (ev) and k-shell (ks), along with the mean number of nodes placed in the top ten by both ExF and eigenvalue centrality, by network family.

	ρ-ev	ρ-ks	overlap
Pareto	0.62	0.79	6.38
Amazon	0.71	0.61	6.96
Internet	0.73	0.82	9.16
Facebook	0.83	0.90	9.35
Astrophysics	0.84	0.92	9.22

Supplementary Table 3. Correlation between expected force and (neighbor) degree. Mean correlation between expected force and the the degree of the seed node, the sum of the degrees of all neighbors to the seed node, and the sum of the degree of all nodes at geodesic distance two from the seed node, taken over fifty networks of 1000 nodes for each class; the correlations are based on the values observed for all network nodes.

	node	neighbors	g-2 neighbors
Pareto	0.79 ± 0.04	0.84 ± 0.02	0.85 ± 0.02
Amazon	0.81 ± 0.05	0.86 ± 0.02	0.83 ± 0.02
Internet	0.64 ± 0.08	0.90 ± 0.01	0.27 ± 0.32
Facebook	0.78 ± 0.04	0.92 ± 0.05	-0.86 ± 0.03
Astrophysics	0.80 ± 0.03	0.94 ± 0.01	-0.83 ± 0.03

Supplementary Table 4. Empirical evidence that fixed multiples of the inverse of the largest eigenvalue give β in the critical range for epidemic take-off. Percentage of nodes with epidemic potential in the interval $[2\%, 98\%]$ when β is set to the given multiple of the $1/\lambda$ (where λ is the largest eigenvalue of the adjacency matrix), by network type. Epidemic potential is measured by simulating 100 spreading processes seeded from the given node and counting how many result in an epidemic. A different multiple is used for each type of spreading process. Denser networks require higher multiples.

	Pareto/Amazon		Internet/Astro/Facebook	
	multiple	percentage	multiple	percentage
SIS-C	4.0	(100/100)%	8.0	(99/99/98)
SIS-D	2.0	(93/89)%	2.5	(83/81/77)
SIR-C	6.0	(100/100)%	8.0	(99/98/96)
SIR-D	3.5	(83/76)%	5.0	(76/78/81)

Supplementary Table 5. Mean correlations between node spreading power metrics and epidemic outcomes on each type of spreading process on the simulated networks, by network model. Shown is the mean and standard error in correlations measured on one hundred networks from each family. This information is duplicated in Figure 2 in the main text. ExF^M is not included for SI models as the modification only makes sense for processes with recovery. Spreading processes are suffixed to indicate simulations in continuous (-C) or discrete (-D) time. Epidemic outcomes are time to half coverage for SI processes and epidemic potential in the remaining processes.

	Expected force	ExF^M	accesibility	eigenvalue centrality	k-shell
Pareto					
SI	0.84 ± 0.04		0.66 ± 0.05	0.38 ± 0.06	0.76 ± 0.05
SIS-C	0.93 ± 0.02	0.89 ± 0.02	0.78 ± 0.05	0.53 ± 0.08	0.77 ± 0.05
SIS-D	0.94 ± 0.02	0.91 ± 0.02	0.78 ± 0.06	0.51 ± 0.09	0.79 ± 0.05
SIR-C	0.91 ± 0.02	0.81 ± 0.03	0.71 ± 0.05	0.43 ± 0.08	0.82 ± 0.03
SIR-D	0.87 ± 0.14	0.78 ± 0.13	0.68 ± 0.12	0.40 ± 0.09	0.82 ± 0.14
Amazon					
SI	0.87 ± 0.02		0.84 ± 0.02	0.44 ± 0.05	0.88 ± 0.02
SIS-C	0.95 ± 0.01	0.92 ± 0.01	0.91 ± 0.02	0.63 ± 0.06	0.72 ± 0.05
SIS-D	0.95 ± 0.01	0.91 ± 0.02	0.92 ± 0.03	0.59 ± 0.06	0.74 ± 0.06
SIR-C	0.92 ± 0.02	0.85 ± 0.02	0.88 ± 0.03	0.53 ± 0.05	0.82 ± 0.04
SIR-D	0.90 ± 0.02	0.81 ± 0.03	0.87 ± 0.04	0.46 ± 0.06	0.87 ± 0.04
Internet					
SI	0.82 ± 0.03		0.77 ± 0.08	0.37 ± 0.04	0.73 ± 0.04
SIS-C	0.92 ± 0.03	0.90 ± 0.01	0.61 ± 0.09	0.65 ± 0.04	0.95 ± 0.01
SIS-D	0.85 ± 0.03	0.96 ± 0.01	0.45 ± 0.08	0.82 ± 0.04	0.89 ± 0.03
SIR-C	0.92 ± 0.02	0.90 ± 0.01	0.62 ± 0.09	0.66 ± 0.04	0.95 ± 0.01
SIR-D	0.89 ± 0.03	0.87 ± 0.02	0.60 ± 0.09	0.60 ± 0.04	0.98 ± 0.01
Astrophysics					
SI	0.81 ± 0.02		0.51 ± 0.07	0.36 ± 0.03	0.6 ± 0.04
SIS-C	0.92 ± 0.01	0.94 ± 0.01	0.31 ± 0.05	0.71 ± 0.02	0.95 ± 0.01
SIS-D	0.85 ± 0.02	0.97 ± 0	0.2 ± 0.04	0.86 ± 0.03	0.96 ± 0.01
SIR-C	0.92 ± 0.01	0.94 ± 0.01	0.31 ± 0.05	0.71 ± 0.02	0.95 ± 0.01
SIR-D	0.89 ± 0.01	0.92 ± 0.01	0.29 ± 0.05	0.67 ± 0.03	0.97 ± 0.01
Facebook					
SI	0.83 ± 0.02		0.43 ± 0.1	0.38 ± 0.02	0.61 ± 0.04
SIS-C	0.9 ± 0.02	0.95 ± 0.01	0.22 ± 0.05	0.73 ± 0.02	0.95 ± 0.01
SIS-D	0.82 ± 0.02	0.97 ± 0	0.14 ± 0.04	0.87 ± 0.02	0.97 ± 0.01
SIR-C	0.9 ± 0.02	0.95 ± 0.01	0.22 ± 0.05	0.73 ± 0.02	0.95 ± 0.01
SIR-D	0.87 ± 0.02	0.94 ± 0.01	0.2 ± 0.05	0.7 ± 0.03	0.97 ± 0.01

Supplementary Table 6. Correlation between spreading power metrics and *tthc* in real world networks. Shown is the estimated correlation from 1,000 nodes on the given network, along with the 95% confidence bounds of the estimate. This information is duplicated in Figure 3 in the main text. The ExF^M is not included here as the modification only makes sense for processes with recovery; an empty column is used to allow easier visual comparison with the remaining tables. Accessibility is not measured for networks with more than 25,000 nodes.

	Expected force	ExF^M	accessibility	eigenvalue centrality	k-shell
PGPgiantcompo	0.69 ± 0.03	–	0.58 ± 0.04	0.19 ± 0.06	0.43 ± 0.05
amazon0302	0.54 ± 0.04	–	–	0.15 ± 0.06	0.30 ± 0.06
amazon0601	0.74 ± 0.03	–	–	0.09 ± 0.06	0.63 ± 0.04
ca-AstroPh	0.84 ± 0.02	–	0.49 ± 0.05	0.29 ± 0.06	0.58 ± 0.04
ca-CondMat	0.84 ± 0.02	–	0.53 ± 0.04	0.26 ± 0.06	0.65 ± 0.04
ca-GrQc	0.78 ± 0.02	–	0.58 ± 0.04	0.16 ± 0.06	0.36 ± 0.05
ca-HepPh	0.82 ± 0.02	–	0.54 ± 0.04	0.20 ± 0.06	0.39 ± 0.05
ca-HepTh	0.78 ± 0.02	–	0.56 ± 0.04	0.05 ± 0.06	0.47 ± 0.05
cit-HepPh	0.82 ± 0.02	–	–	0.28 ± 0.06	0.68 ± 0.03
cit-HepTh	0.84 ± 0.02	–	0.57 ± 0.04	0.38 ± 0.05	0.64 ± 0.04
com-dblp	0.79 ± 0.02	–	–	0.05 ± 0.06	0.36 ± 0.05
email-EuAll	0.41 ± 0.05	–	–	0.34 ± 0.05	0.50 ± 0.05
email-Uni	0.92 ± 0.01	–	0.61 ± 0.04	0.56 ± 0.04	0.84 ± 0.02
facebooklcc	0.86 ± 0.02	–	–	0.19 ± 0.06	0.59 ± 0.04
loc-brightkite	0.79 ± 0.02	–	–	0.13 ± 0.06	0.54 ± 0.04
loc-gowalla	0.66 ± 0.03	–	–	0.25 ± 0.06	0.53 ± 0.04
p2p-Gnutella31	0.94 ± 0.01	–	0.72 ± 0.03	0.53 ± 0.04	0.92 ± 0.01
soc-Epinions1	0.80 ± 0.02	–	–	0.33 ± 0.06	0.47 ± 0.05
soc-Slashdot0902	0.84 ± 0.02	–	–	0.42 ± 0.05	0.60 ± 0.04
soc-sign-epinions	0.81 ± 0.02	–	–	0.29 ± 0.06	0.47 ± 0.05
web-Google	0.69 ± 0.02	–	–	0.07 ± 0.04	0.59 ± 0.02
web-NotreDame	0.43 ± 0.05	–	–	0.18 ± 0.06	0.26 ± 0.06
web-Stanford	0.25 ± 0.06	–	–	0.06 ± 0.06	0.12 ± 0.06
wiki-Vote	0.86 ± 0.02	–	0.50 ± 0.05	0.50 ± 0.05	0.72 ± 0.03

Supplementary Table 7. Correlation between spreading power metrics and epidemic potential in discrete time SIS processes on real world networks. Shown is the estimated correlation from 1,000 nodes on the given network, along with the 95% confidence bounds of the estimate. This information is duplicated in Figure 3 in the main text. Accessibility is not measured for networks with more than 25,000 nodes.

	Expected force	ExF^M	accessibility	eigenvalue centrality	k-shell
PGPgiantcompo	0.87 ± 0.02	0.91 ± 0.01	0.62 ± 0.04	0.33 ± 0.06	0.77 ± 0.03
amazon0302	0.79 ± 0.02	0.80 ± 0.02	–	0.12 ± 0.06	0.51 ± 0.05
amazon0601	0.77 ± 0.03	0.87 ± 0.02	–	-0.01 ± 0.06	0.68 ± 0.03
ca-AstroPh	0.94 ± 0.01	0.92 ± 0.01	0.51 ± 0.05	0.41 ± 0.05	0.83 ± 0.02
ca-CondMat	0.93 ± 0.01	0.92 ± 0.01	0.59 ± 0.04	0.35 ± 0.05	0.85 ± 0.02
ca-GrQc	0.92 ± 0.01	0.92 ± 0.01	0.52 ± 0.05	0.25 ± 0.06	0.7 ± 0.03
ca-HepPh	0.92 ± 0.01	0.90 ± 0.01	0.51 ± 0.05	0.28 ± 0.06	0.55 ± 0.04
ca-HepTh	0.92 ± 0.01	0.93 ± 0.01	0.64 ± 0.04	0.10 ± 0.06	0.72 ± 0.03
cit-HepPh	0.93 ± 0.01	0.94 ± 0.01	0.55 ± 0.04	0.38 ± 0.05	0.93 ± 0.01
cit-HepTh	0.93 ± 0.01	0.93 ± 0.01	0.71 ± 0.03	0.57 ± 0.04	0.90 ± 0.01
com-dblp	0.90 ± 0.01	0.55 ± 0.03	–	0.08 ± 0.04	0.55 ± 0.03
email-EuAll	0.36 ± 0.05	0.9 ± 0.01	–	0.64 ± 0.04	0.85 ± 0.02
email-Uni	0.95 ± 0.01	0.97 ± 0.00	0.61 ± 0.04	0.75 ± 0.03	0.97 ± 0.00
facebooklcc	0.93 ± 0.01	0.94 ± 0.01	–	0.31 ± 0.06	0.88 ± 0.01
loc-brightkite	0.85 ± 0.02	0.95 ± 0.01	0.58 ± 0.04	0.29 ± 0.06	0.85 ± 0.02
loc-gowalla	0.68 ± 0.03	0.91 ± 0.01	–	0.51 ± 0.05	0.89 ± 0.01
p2p-Gnutella31	0.95 ± 0.01	0.97 ± 0.00	0.83 ± 0.02	0.68 ± 0.03	0.92 ± 0.01
soc-Epinions1	0.77 ± 0.03	0.96 ± 0.01	–	0.63 ± 0.04	0.85 ± 0.02
soc-Slashdot0902	0.80 ± 0.02	0.97 ± 0.00	–	0.71 ± 0.03	0.93 ± 0.01
soc-sign-epinions	0.76 ± 0.03	0.96 ± 0.01	–	0.54 ± 0.04	0.81 ± 0.02
web-Google	0.79 ± 0.02	0.93 ± 0.01	–	0.10 ± 0.06	0.91 ± 0.01
web-NotreDame	0.73 ± 0.03	0.92 ± 0.01	–	0.34 ± 0.06	0.49 ± 0.05
web-Stanford	0.70 ± 0.03	0.89 ± 0.01	–	0.38 ± 0.05	0.77 ± 0.03
wiki-Vote	0.94 ± 0.01	0.96 ± 0.01	0.48 ± 0.05	0.71 ± 0.03	0.95 ± 0.01

Supplementary Table 8. Correlation between spreading power metrics and epidemic potential in discrete time SIR processes on real world networks. Shown is the estimated correlation from 1,000 nodes on the given network, along with the 95% confidence bounds of the estimate. This information is duplicated in Figure 3 in the main text. Accessibility is not measured for networks with more than 25,000 nodes.

	Expected force	ExF^M	accessibility	eigenvalue centrality	k-shell
PGPgiantcompo	0.82 ± 0.02	0.86 ± 0.02	0.69 ± 0.03	0.36 ± 0.05	0.73 ± 0.03
amazon0302	0.67 ± 0.03	0.65 ± 0.04	–	0.19 ± 0.06	0.33 ± 0.06
amazon0601	0.75 ± 0.03	0.83 ± 0.02	–	-0.03 ± 0.06	0.71 ± 0.03
ca-AstroPh	0.94 ± 0.01	0.93 ± 0.01	0.53 ± 0.04	0.42 ± 0.05	0.84 ± 0.02
ca-CondMat	0.92 ± 0.01	0.91 ± 0.01	0.65 ± 0.04	0.38 ± 0.05	0.83 ± 0.02
ca-GrQc	0.85 ± 0.02	0.83 ± 0.02	0.65 ± 0.04	0.27 ± 0.06	0.55 ± 0.04
ca-HepPh	0.91 ± 0.01	0.91 ± 0.01	0.53 ± 0.04	0.31 ± 0.06	0.59 ± 0.04
ca-HepTh	0.89 ± 0.01	0.88 ± 0.01	0.70 ± 0.03	0.10 ± 0.06	0.64 ± 0.04
cit-HepPh	0.93 ± 0.01	0.94 ± 0.01	0.56 ± 0.04	0.39 ± 0.05	0.94 ± 0.01
cit-HepTh	0.93 ± 0.01	0.92 ± 0.01	0.70 ± 0.03	0.54 ± 0.04	0.87 ± 0.01
com-dblp	0.89 ± 0.01	0.55 ± 0.03	–	0.08 ± 0.04	0.53 ± 0.03
email-EuAll	0.31 ± 0.06	0.87 ± 0.02	–	0.60 ± 0.04	0.81 ± 0.02
email-Uni	0.94 ± 0.01	0.97 ± 0.00	0.63 ± 0.04	0.81 ± 0.02	0.96 ± 0.00
facebooklcc	0.93 ± 0.01	0.95 ± 0.01	–	0.32 ± 0.06	0.89 ± 0.01
loc-brightkite	0.84 ± 0.02	0.95 ± 0.01	0.61 ± 0.04	0.31 ± 0.06	0.87 ± 0.02
loc-gowalla	0.68 ± 0.03	0.90 ± 0.01	–	0.47 ± 0.05	0.86 ± 0.02
p2p-Gnutella31	0.96 ± 0.00	0.97 ± 0.00	0.84 ± 0.02	0.68 ± 0.03	0.94 ± 0.01
soc-Epinions1	0.77 ± 0.03	0.96 ± 0.01	–	0.64 ± 0.04	0.85 ± 0.02
soc-Slashdot0902	0.82 ± 0.02	0.96 ± 0.00	–	0.69 ± 0.03	0.91 ± 0.01
soc-sign-epinions	0.76 ± 0.03	0.96 ± 0.01	–	0.54 ± 0.04	0.81 ± 0.02
web-Google	0.79 ± 0.02	0.91 ± 0.01	–	0.10 ± 0.06	0.86 ± 0.02
web-NotreDame	0.61 ± 0.04	0.80 ± 0.02	–	0.31 ± 0.06	0.44 ± 0.05
web-Stanford	0.68 ± 0.03	0.78 ± 0.02	–	0.37 ± 0.05	0.65 ± 0.04
wiki-Vote	0.93 ± 0.01	0.96 ± 0.00	0.48 ± 0.05	0.71 ± 0.03	0.95 ± 0.01

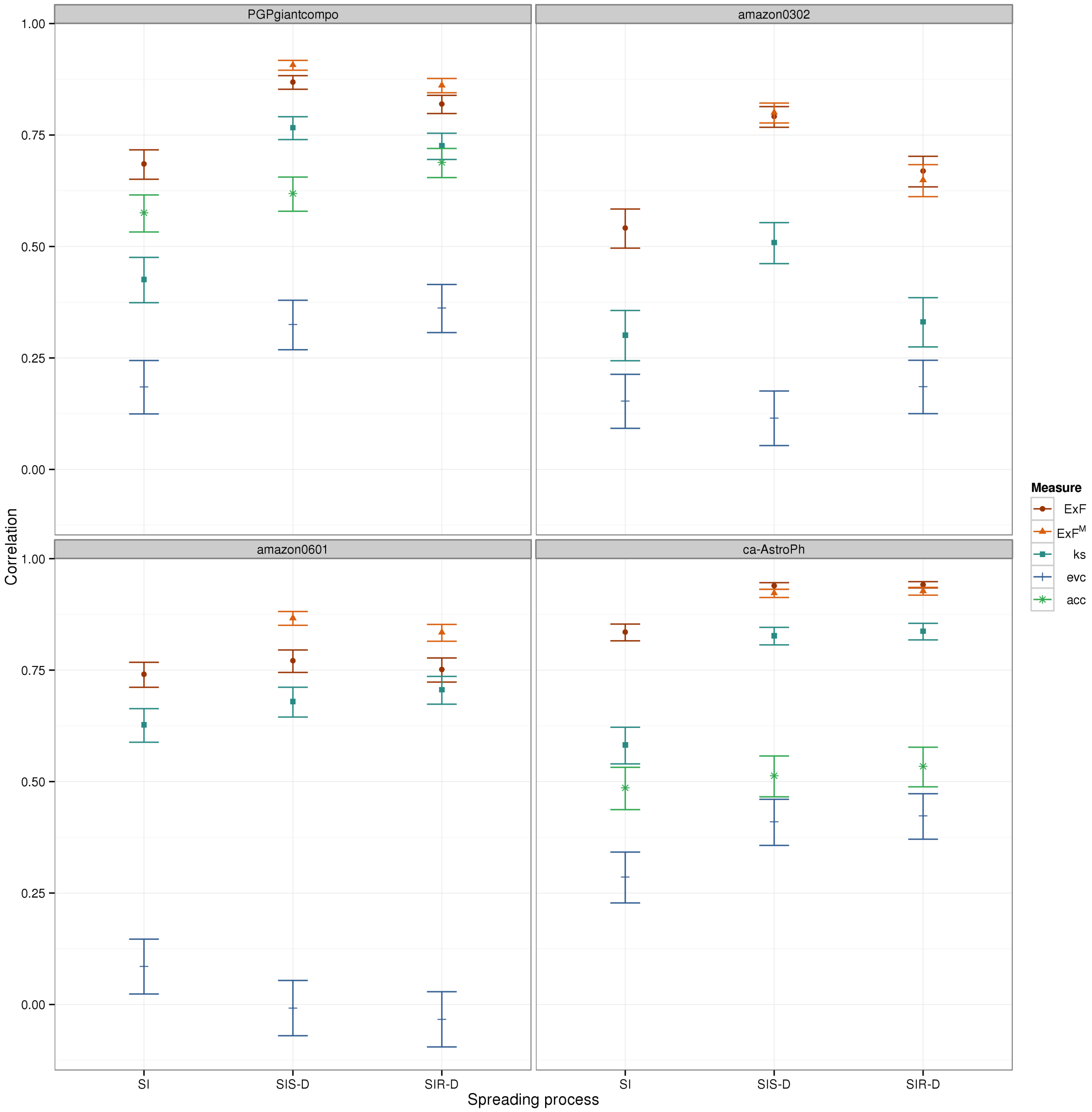

Supplementary Figure 1. Correlation of spreading power metrics to epidemic outcomes on real networks, detailed view. Larger versions of the point and error bar plots from Figure 3, Main text, showing the observed correlation and 95% confidence interval between each measure and spreading process outcome on the real networks. The expected force and ExF^M (orange shades) show strong performance, consistently outperforming the other metrics (k-shell, eigenvalue centrality, and accessibility when computed, blue-green shades). The epidemic outcome for SI processes is the time until half the network is infected. For SIS and SIR processes it is the probability that an epidemic is observed. The suffix "-D" indicates spreading processes simulated in discrete time.

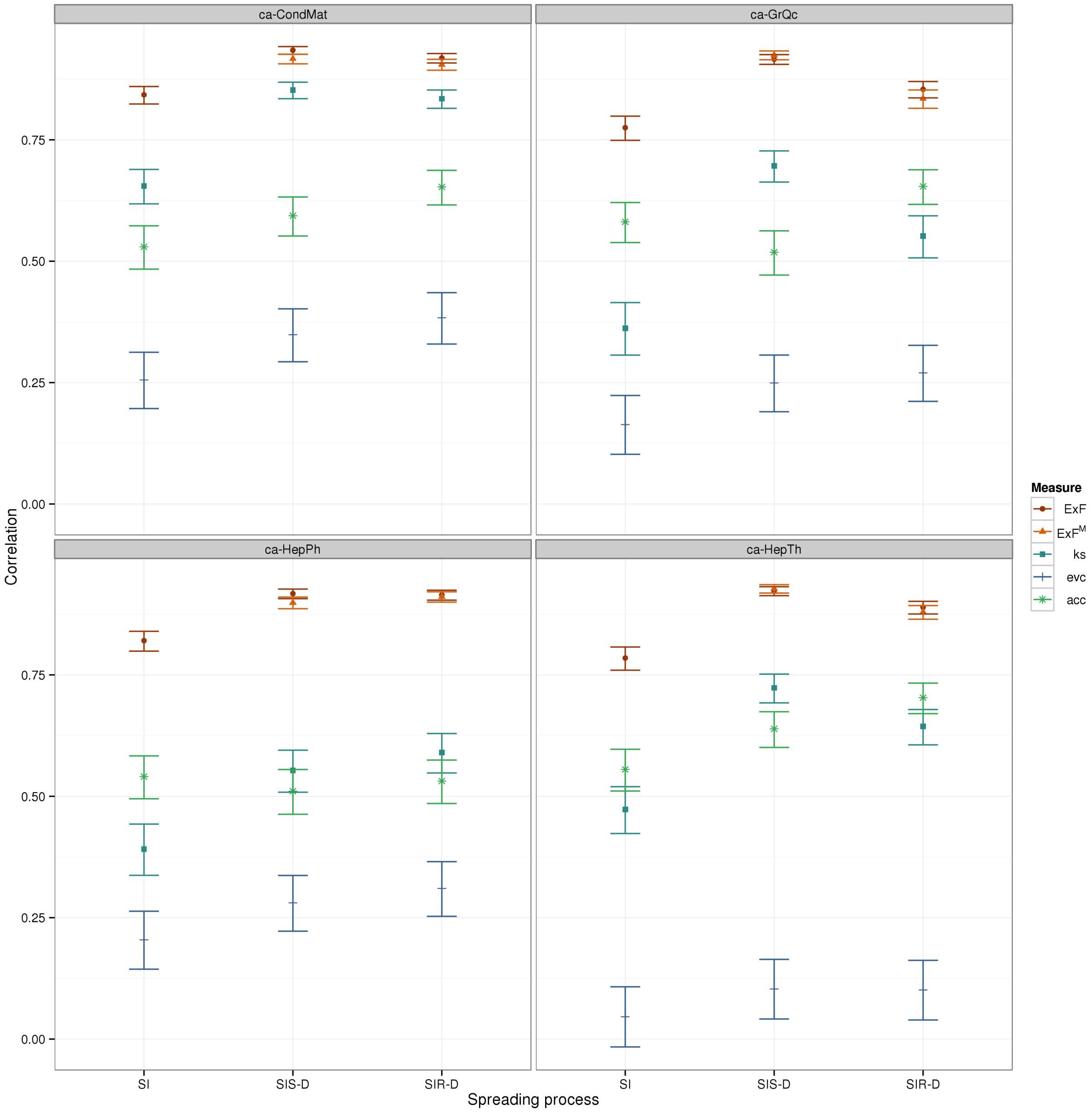

Supplementary Figure 2. Correlation of spreading power metrics to epidemic outcomes on real networks, detailed view. Larger versions of the point and error bar plots from Figure 3, Main text, showing the observed correlation and 95% confidence interval between each measure and spreading process outcome on the real networks. The expected force and ExF^M (orange shades) show strong performance, consistently outperforming the other metrics (k-shell, eigenvalue centrality, and accessibility when computed, blue-green shades). The epidemic outcome for SI processes is the time until half the network is infected. For SIS and SIR processes it is the probability that an epidemic is observed. The suffix "-D" indicates spreading processes simulated in discrete time.

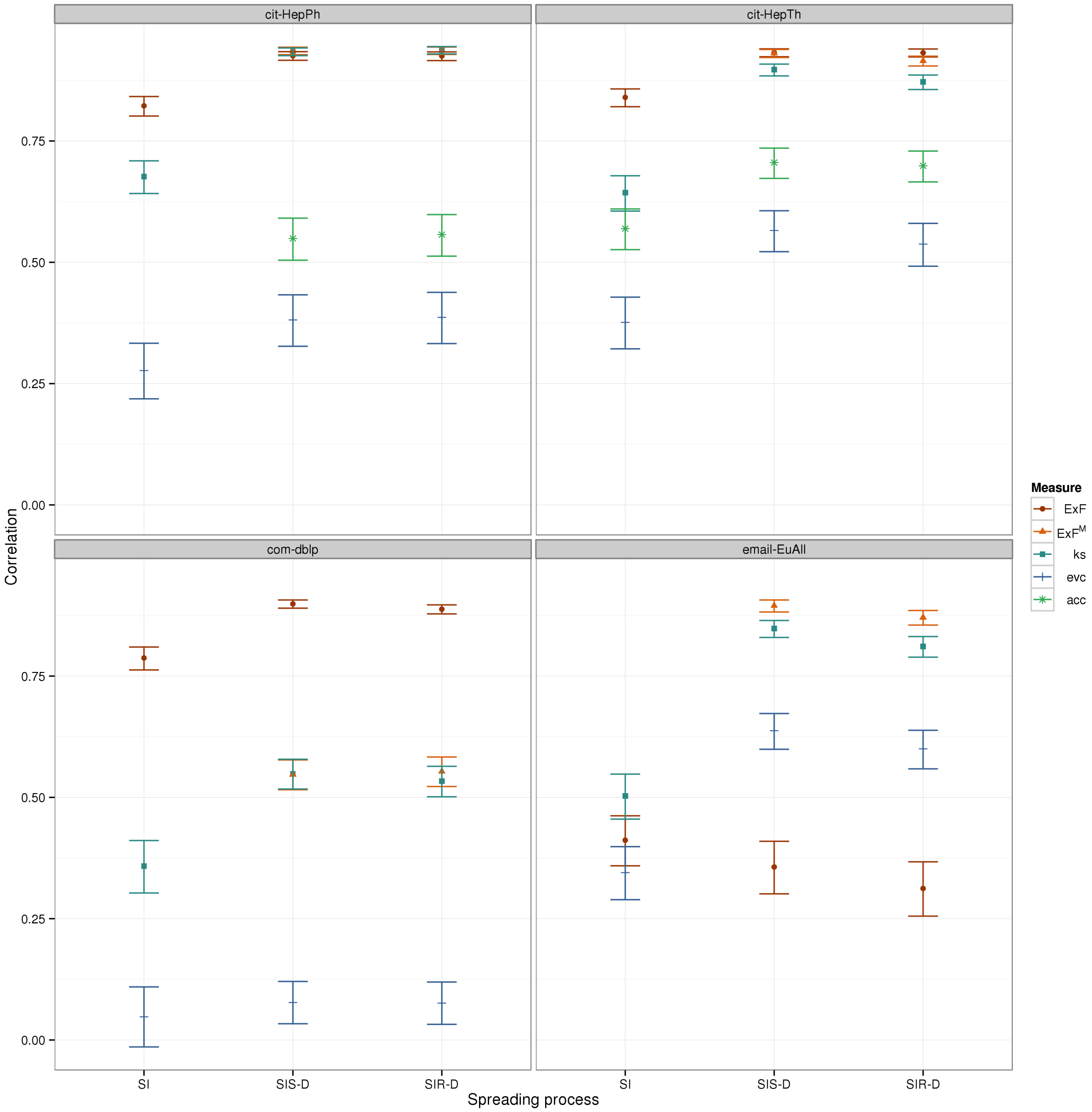

Supplementary Figure 3. Correlation of spreading power metrics to epidemic outcomes on real networks, detailed view. Larger versions of the point and error bar plots from Figure 3, Main text, showing the observed correlation and 95% confidence interval between each measure and spreading process outcome on the real networks. The expected force and ExF^M (orange shades) show strong performance, consistently outperforming the other metrics (k-shell, eigenvalue centrality, and accessibility when computed, blue-green shades). The epidemic outcome for SI processes is the time until half the network is infected. For SIS and SIR processes it is the probability that an epidemic is observed. The suffix "-D" indicates spreading processes simulated in discrete time.

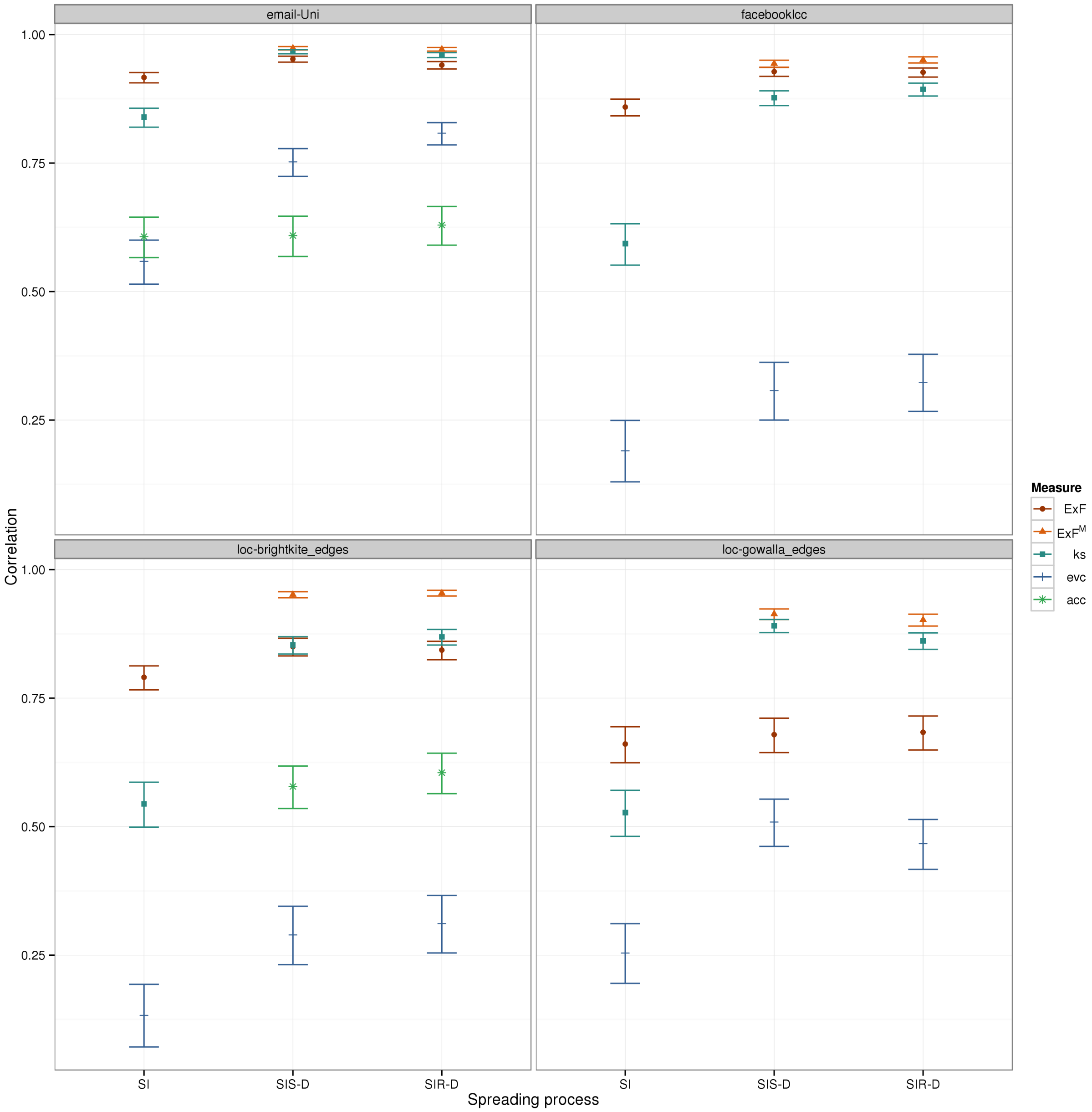

Supplementary Figure 4. Correlation of spreading power metrics to epidemic outcomes on real networks, detailed view. Larger versions of the point and error bar plots from Figure 3, Main text, showing the observed correlation and 95% confidence interval between each measure and spreading process outcome on the real networks. The expected force and ExF^M (orange shades) show strong performance, consistently outperforming the other metrics (k-shell, eigenvalue centrality, and accessibility when computed, blue-green shades). The epidemic outcome for SI processes is the time until half the network is infected. For SIS and SIR processes it is the probability that an epidemic is observed. The suffix "-D" indicates spreading processes simulated in discrete time.

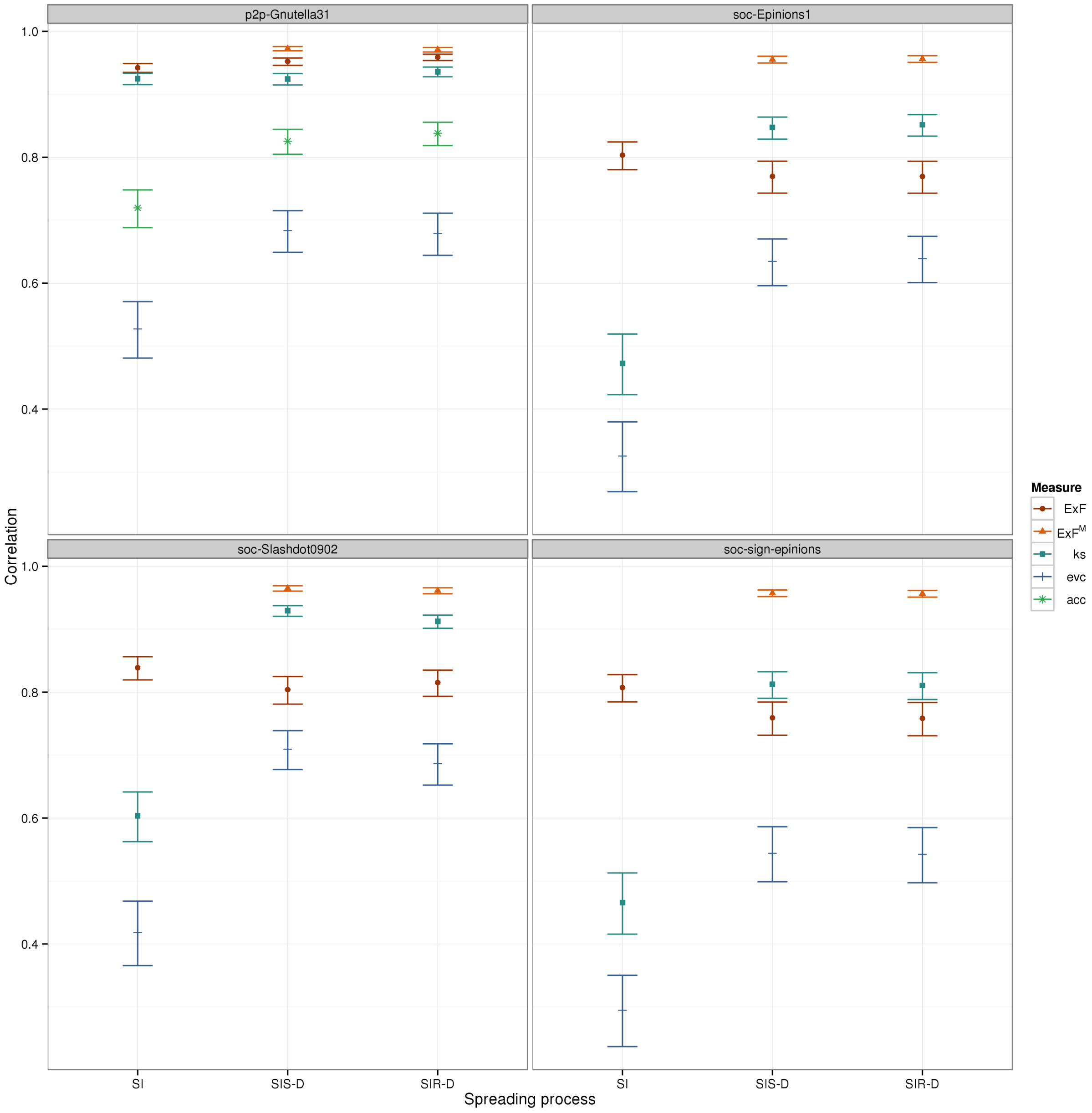

Supplementary Figure 5. Correlation of spreading power metrics to epidemic outcomes on real networks, detailed view. Larger versions of the point and error bar plots from Figure 3, Main text, showing the observed correlation and 95% confidence interval between each measure and spreading process outcome on the real networks. The expected force and ExF^M (orange shades) show strong performance, consistently outperforming the other metrics (k-shell, eigenvalue centrality, and accessibility when computed, blue-green shades). The epidemic outcome for SI processes is the time until half the network is infected. For SIS and SIR processes it is the probability that an epidemic is observed. The suffix "-D" indicates spreading processes simulated in discrete time.

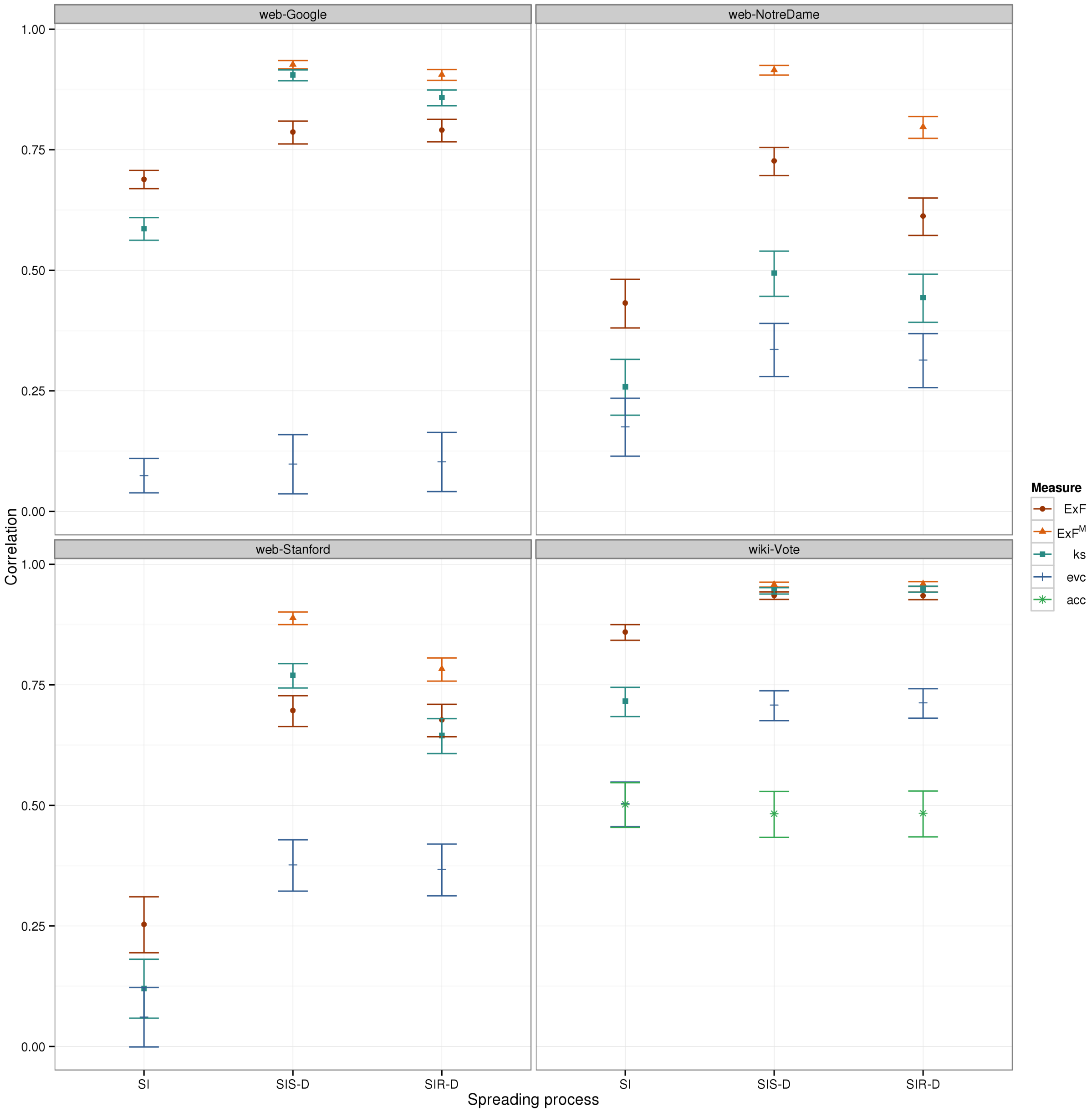

Supplementary Figure 6. Correlation of spreading power metrics to epidemic outcomes on real networks, detailed view. Larger versions of the point and error bar plots from Figure 3, Main text, showing the observed correlation and 95% confidence interval between each measure and spreading process outcome on the real networks. The expected force and ExF^M (orange shades) show strong performance, consistently outperforming the other metrics (k-shell, eigenvalue centrality, and accessibility when computed, blue-green shades). The epidemic outcome for SI processes is the time until half the network is infected. For SIS and SIR processes it is the probability that an epidemic is observed. The suffix "-D" indicates spreading processes simulated in discrete time.
